# Exploring gender differences in knowledge and practices related to antibiotic use in Southeast Asia: A scoping review

**DOI:** 10.1371/journal.pone.0259069

**Published:** 2021-10-26

**Authors:** Phuc Pham-Duc, Kavitha Sriparamananthan

**Affiliations:** 1 Center for Public Health and Ecosystem Research, Hanoi University of Public Health, Hanoi, Vietnam; 2 Institute of Environmental Health and Sustainable Development, Hanoi, Vietnam; 3 Veterinarians Without Borders/ Vétérinaires Sans Frontières, Ottawa, Ontario, Canada; University of Lincoln, UNITED KINGDOM

## Abstract

Inappropriate use of antibiotics has been one of the main contributors to antimicrobial resistance, particularly in Southeast Asia. Different genders are prone to different antibiotic use practices. The objective of this scoping review is to understand the extent and type of evidence available on gender differences in antibiotic use across Southeast Asia. The search strategy for this scoping review involved PubMed, Semantic Scholar, BioMed Central and ProQuest. Two-level screening was applied to identify the final sample of relevant sources. Thematic content analysis was then conducted on the selected final sources to identify recurring themes related to gender differences in antibiotic use and a narrative account was developed based on the themes. Recommendations for next steps regarding reducing inappropriate antibiotic use and gender considerations that need to be made when developing future interventions were also identified. Research on gender and antibiotic use remains scarce. Studies that discuss gender within the context of antibiotic use often mention differences between males and females in knowledge, attitudes and/or behaviour, however, do not explore reasons for these differences. Gender differences in antibiotic use were generally examined in terms of: (i) knowledge of antibiotic use and antimicrobial resistance and (ii) practices related to antibiotic use. Evidence indicated that differences between males and females in knowledge and practices of antibiotic use varied greatly based on setting. This indicates that gender differences in antibiotic use are greatly contextual and intersect with other sociodemographic factors, particularly education and socioeconomic status. Educational interventions that are targeted to meet the specific needs of males and females and delivered through pharmacists and healthcare professionals were the most common recommendations for reducing inappropriate use of antibiotics in the community. Such targeted interventions require further qualitative research on factors influencing differences in knowledge and practices related to antibiotic use among males and females. In addition, there is also a need to strengthen monitoring and regulation practices to ensure accessibility to affordable, quality antibiotics through trusted sources.

## Introduction

Antimicrobial resistance (AMR) is currently a growing global threat to human, animal and environmental health [1, 2]. Antibiotics are one of the most commonly used medicines and inappropriate use of antibiotics is one of the main causes of AMR [3, 4]. It is particularly a challenge in regions such as Southeast Asia which are highly populated and burdened with infectious diseases [5]. Although antibiotics can reduce the burden of infectious diseases, inappropriate use leads to a decreased ability to treat bacterial infections, thus fostering the risk of increased morbidity and mortality due to the spread of resistant bacteria [2]. Extensive inappropriate use of antibiotics in animals can also result in the spread of antibiotic resistant bacteria to humans through direct contact, consumption of meat or indirect environmental pathways [1]; thus, creating a vicious cycle affecting humans, animals and the environment. There remains a need for effective interventions to reduce inappropriate antibiotic use [4]. Such interventions require examination of factors that influence antibiotic use [6].

A study analyzing national action plans on AMR in Southeast Asia highlighted the importance of examining demographic factors such as gender that may put already vulnerable groups at greater risk of AMR [6]. The national action plans of ten countries belonging to the Association of Southeast Asia Nations (ASEAN), namely Brunei, Cambodia, Indonesia, Laos, Malaysia, the Philippines, Singapore, Thailand and Vietnam, were examined. Gender considerations were not mentioned in any of the national action plans [6]. Influencing human behaviour to reduce inappropriate use of antibiotics is complex and in order to effectively combat inappropriate use, the influence of gender must be taken into consideration [6].

Furthermore, studies examining antibiotic use and AMR at the community level remain limited, with those examining gender differences even more sparse. There is limited research and literature on gender differences in antibiotic use and even less examining how gender specific factors such gender norms, roles and relations within a community affect antibiotic use [7]. The few existing studies that have found gender related patterns and differences in antibiotic use emphasize that in order to improve the effectiveness of strategies to reduce inappropriate antibiotic use and AMR, gender must be examined, specifically at the community level [7]. Examining and integrating gender will not only help strengthen AMR research and surveillance but will also help identify areas for potential partnerships and promote engagement with various stakeholders from different sectors relevant to tackling AMR [7].

A preliminary search of MEDLINE, PubMed, the Cochrane Database of Systematic Reviews and JBI Evidence Synthesis was conducted to identify whether there are any existing systematic or scoping reviews on the topic. A few systematic reviews examining antibiotic use were identified [8, 9]; however, gender specific behaviour was not the primary focus of any of these reviews. Although one review focusing on self-medication with antibiotics in Southeast Asia did mention that males were more likely to self-medicate than females, it did not explore or discuss this further [10]. Thus, to the best of our knowledge, there are no existing reviews focusing primarily on the examination of the role of gender in antibiotic use at the community level.

This scoping review will help address this gap in research by mapping out studies that exist on this topic, and thus serve as a starting point for further research. It examines the role of gender in antibiotic use by exploring gender differences in antibiotic knowledge and practices in Southeast Asia. The findings will inform the integration of gender into AMR research and surveillance strategies in Southeast Asia, and more specifically in Vietnam.

One Health focuses on achieving optimal health outcomes through a transdisciplinary, collaborative approach that examines the intersectionality and interdependent nature of human, animal and environmental health [11]. AMR is especially of concern in low- and middle-income countries due to limited optimal water and sanitation, health and food systems as well as a high burden of infectious diseases [6]. Successful tackling of AMR thus requires coordination and collaboration across regional, national and local levels and between animal, human and environmental health sectors through a One Health Approach [6]. In order to apply a One Health lens, articles discussing antibiotic use in animals, humans and the environment were examined for this review. In other words, it includes studies examining antibiotic use in households and on farms.

## Materials and methods

The methodology of the scoping review was developed in accordance with the Joanna Briggs Institute methodology for scoping reviews [12]. The review included the following steps: (i) identifying the research question, (ii) developing a search strategy, (iii) study selection and (iv) data analysis and presentation. The Preferred Reporting Items for Systematic Reviews and Meta-Analyses (PRISMA) checklist was also used to guide the reporting quality of this study. A review protocol was developed for internal use only.

### Identifying the research question

A preliminary investigation on gender, AMR and Southeast Asia was conducted to examine existing research and identify opportunities for further exploration. Based on this preliminary investigation, primary and secondary research questions were developed. The scoping review specifically aimed to address the following questions:

#### Primary review question

What is known from existing literature about gender differences in antibiotic use in Southeast Asia?

#### Secondary review questions

1. What gender differences in antibiotic use exist across Southeast Asia?
2. What models exist to examine the role of gender in antibiotic use in Southeast Asia?

### Search strategy

#### Eligibility criteria

Relevant elements of the population, concepts and context (PCC) framework were applied to the research question to identify key terms [12]. The population element was not relevant for this scoping review as this review did not focus on a specific cohort or condition. The core concept examined in this review is antibiotic use. This includes antibiotic use in humans, animals and plants in order to ensure application of a One Health lens. Also, this review only included studies that examined gender in the context of antibiotic use. Studies that either focus on all genders or a specific gender were included in this review. The scoping review only included studies within the context of Southeast Asia to ensure relevancy of the information to Vietnam. The PCC framework used to inform the search strategy is presented in Table 1.

**Table 1:** PCC framework for search strategy development.

Framework	Element	Key Terms
PCC	Population	n/a
	Concept	antibiotic use
	Context	gender
		Southeast Asia

A pilot search was conducted in MEDLINE and PubMed databases to identify articles on the topic. The identified key terms were combined to develop the search phrases used for the pilot search. Search phrases included “gender AND antibiotic use”, “gender AND antibiotic use AND Southeast Asia” and “antibiotic use AND Southeast Asia”. The text words contained in the titles and abstracts of relevant articles were examined to identify synonyms for the key terms. An additional search was then conducted in PubMed to identify Medical Subject Headings (MESH).

The final searches were as follows: “gender AND antibiotic use AND Southeast Asia” and “gender AND antibiotic use OR antibiotic resistance OR knowledge AND attitudes AND practice AND Southeast Asia.”

Searches were also conducted using the following phrases and terminology for each of the countries in the region of interest, more specifically, member countries of the Association of Southeast Asian Nations (ASEAN), as follows: “gender AND antibiotic use AND Vietnam,” “gender AND antibiotic use AND Malaysia,” and “gender AND antibiotic use AND Thailand,” to name a few. However, due to the limited number of relevant search results, the search was widened to be “gender AND antibiotic use AND Southeast Asia.”

Similarly, searches were conducted using the following phrases “gender AND antibiotic use OR antibiotic resistance OR AMR OR knowledge AND attitudes AND practice AND Vietnam, etc.” for each of the ASEAN countries. This search was also widened to include Southeast Asia due to the limited number of relevant results that appeared when each specific country was used in the search. Moreover, a separate search was conducted by replacing the term “antibiotic resistance” with “AMR.” In PubMed, it was found that both searches yielded the same results. However, in ProQuest and Semantic Scholar, using the term “antibiotic resistance” yielded more results, including the results from the use of the term “AMR,” and thus, antibiotic resistance was used in the final search strategy to widen the results.

#### Inclusion criteria

The review only included articles that were: (i) peer-reviewed journal articles; (ii) published in English; (iii) published in the last ten years (from 2011 to 2021); and (iv) focused on Southeast Asia.

Only peer reviewed articles were included to ensure validity of the findings. In addition, articles published in the last ten years were of focus to ensure relevancy to current antibiotic use practices and AMR surveillance strategies. Articles that either focused on Southeast Asia as a whole or specific countries within the region were included. More specifically, only articles on member countries of ASEAN, namely Brunei Darussalam, Cambodia, Indonesia, Lao PDR, Malaysia, Myanmar, Philippines, Singapore, Thailand and Vietnam [13], were of interest as findings from these countries would be of most relevance to the context of Vietnam.

#### Exclusion criteria

Articles focusing only on healthcare settings such as clinics or hospitals were excluded as this review focuses on antibiotic use in the community. Although antibiotic use in healthcare settings also influences AMR, research examining antibiotic use in community settings is scarcer. There is a need to examine use in community settings to develop a better understanding of what influences antibiotic use behaviour, especially use without consultation at healthcare settings. Thus, this review aimed to inform this gap in research and specifically map out articles that examine gender differences in antibiotic use in the community setting. Identifying root causes to inappropriate use at the community level will also inform practice in healthcare settings as well. Moreover, articles examining gender in the context of antibiotic prescription were excluded. This was because preliminary investigation of the topic indicated that articles examining differences in gender and prescribing patterns focused on primary care settings and prescribing behaviour of healthcare professionals as opposed to antibiotic use behaviour among the general public.

### Identifying relevant studies

Articles were sourced from the following databases: PubMed, Semantic Scholar, BioMed Central (BMC) and ProQuest. Two searches were run in each database, one for each of the aforementioned final searches, except in ProQuest. In ProQuest, the search “gender AND antibiotic use OR antibiotic resistance OR knowledge AND attitudes AND Practice AND Southeast Asia” was too broad and presented over 100,000 results. Thus, this search was excluded. Although MEDLINE was used in the pilot search, it was not used as one of the final databases to run the searches as MEDLINE is the main part of PubMed, which was included. The date last searched was June 25, 2021.

### Study selection

Following the search, all identified citations were collated and uploaded in Mendeley Version 1.19.8 software and duplicates removed. Two-level screening was then applied to identify relevant articles. Prior to screening all identified citations, a pilot test was conducted using the inclusion and exclusion criteria to determine whether the criteria clearly captured articles relevant for this review. First, titles and abstracts were screened, and potentially relevant sources identified. These sources were then retrieved in full and assessed in detail using the inclusion and exclusion criteria. Reasons for exclusion of sources were recorded. Mendeley Version 1.19.8 software was used to consolidate, review and annotate the final sample of included sources.

**Fig 1:**
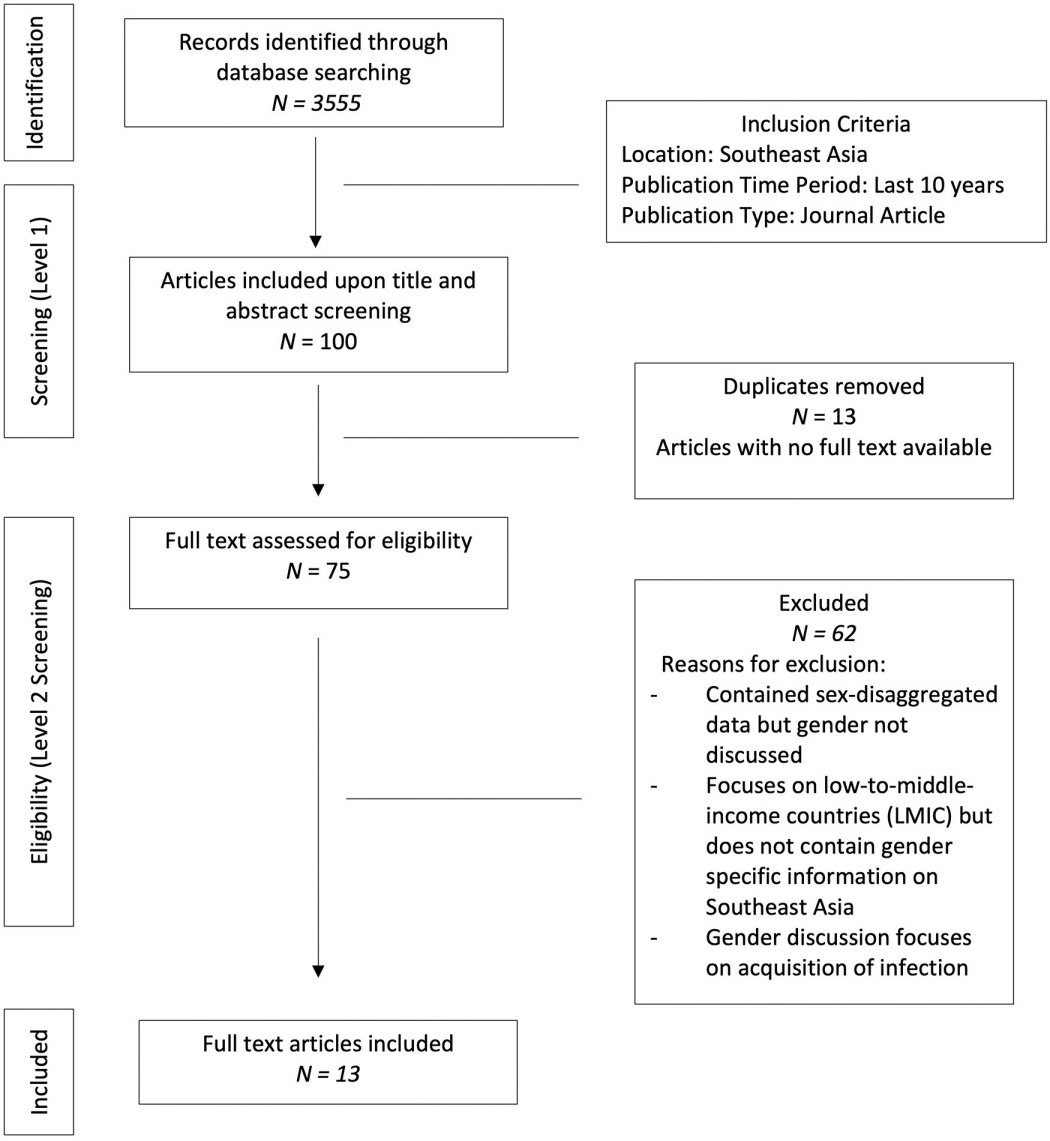
PRISMA-ScR diagram of source selection process.

Upon comprehensive screening of the titles and abstracts of all search results in each of the databases, a full text screening of selected articles was conducted to identify the final set of articles for inclusion. A Preferred Reporting Items for Systematic Reviews and Meta-Analyses extension for scoping review (PRISMA-ScR) diagram detailing the selection process is provided (Fig 1).

### Data charting

A data chart was developed with relevant components to address the research questions. As each included article in the final sample was analyzed, the relevant data was input into the data chart. The data chart included the following information: author(s), country of origin (where the study was conducted), aim, study population and sample size, research design and gender specific key findings and recommendations. The data chart was used for data analysis and informed the development of relevant themes to address the research questions.

### Data analysis

The charted information from the final sample of included articles was examined using thematic content analysis. The key findings from the data chart were examined for patterns, coded and recurring themes identified. These major themes were then examined critically to identify their relationship to the research questions of this review. The recommendations of the included articles were also analyzed in the same manner to identify areas for further research and to inform future interventions addressing inappropriate antibiotic use.

The final sources included in this review were examined in detail to identify any biases and determine whether measures were taken to ensure validity and reliability. Most of the studies had taken measures to ensure validity and/or reliability. Despite the biases outlined in the individual studies, the risk of these biases on the findings outlined in this paper are minimal as this review primarily aimed to identify and map out existing research on the topic and serve as a starting point for further research. The impact of these individual studies on the conclusion of this scoping review is discussed in more detail in a latter part of this paper.

## Results and discussion

### Study selection

A total of 3555 articles resulted from the initial search. Upon screening of the titles and abstracts of these articles, 100 articles remained. For articles which focused on a specific disease and did not include the information on antibiotics or gender in the title or abstract, a quick scan of the entire source was done to determine whether it should be included. Of the 100 articles which remained upon level one of screening, 13 duplicates were removed and full text was not available for 12 sources. Thus, 75 articles remained and were examined for eligibility (level two of screening). As outlined in Fig 1, 13 of the 75 articles were included in this review. The remaining 62 were excluded as they did not discuss gender with regards to antibiotic use or did not have enough information to address the research questions of this review within the context of Southeast Asia.

### Study characteristics

A majority of the 13 articles included in the review were conducted in Malaysia and Vietnam. The remaining were conducted in Cambodia, the Philippines, and Thailand. One article focused on Southeast Asia as a whole. No relevant studies were available from Brunei Darussalam, Indonesia, Lao PDR, Myanmar and Singapore in particular.

All included articles were published between the period of 2013 to 2020. Samples examined in the studies included adults who had experience with antibiotic consumption, households with farms, small- and medium-scale producers, children from primary school and fathers of children who either had pneumonia or pneumonia-like symptoms. Key characteristics of all 13 sources included in this review are detailed in the data chart presented in Table 2.

**Table 2:** Data chart with key characteristics of included sources.

Author(s) (Year) [Ref]	Country of origin	Aim	Study population and sample size	Research design	Gender Specific key findings and recommendations
Choo, et al. (2018) [14]	Malaysia	To explore beliefs, knowledge, and practice on antibiotic use among public and determine the population characteristics associated with inappropriate use of antibiotics	2632 adults ≥18 years, literate, and had experience in antibiotics consumption	Cross-sectional study Close-ended self-administered questionnaires	Respondent’s gender had significant association with belief, knowledge, and practice. Practice: Male respondents, under the age of 60, with non-tertiary level education, who had not heard of antibiotic resistance, were more likely to use leftover antibiotics and not complete the course. Belief: Males were more likely to believe that antibiotics should be stopped when they felt better and that their health in the future depends on antibiotics. Knowledge: Males were more likely to have inappropriate knowledge on adherence to course of antibiotics and leftovers. *Recommendations*: more targeted educational interventions on indication of antibiotic use and antibiotic resistance further qualitative research on reasons of inappropriate antibiotic use
Carrique-Mas, et al. (2015) [1]	Vietnam	(i) To describe and quantify levels of antimicrobial usage in farms in the Mekong Delta (ii) To identify factors associated with usage	208 chicken farms stratified by size (10–200 chickens; >200–2000 chickens)	Survey questionnaires with open and closed questions	Farms run by females used less amounts of antimicrobials. Farms run by males had higher levels of usage per week per chicken. *Recommendations*: Training of household farmers on the correct administration of antimicrobials The finding that female farmers used less antimicrobials requires further investigation
Strom, et al. (2018) [5]	Cambodia	To obtain information on knowledge, attitudes and practices related to antimicrobial use in small-scale pig farming in Phnom Penh, and data on phenotypic AMR in Escherichia coli isolated from pigs kept by the farming households.	91 small-scale urban and peri-urban pig farms (family farm conditions)	Cross-sectional study Semi-structured questionnaire	At 66% of farms, male household heads were responsible for treating sick animals while female household heads were responsible for treating sick animals at only 16% of farms. 74% of males in comparison to 39% of females reported that they had heard of AMR. This may be due to men generally attaining higher levels of education or that men were usually responsible for treating sick pigs and thus, had more discussions with veterinarians. *Recommendations*: professional animal health systems that involve medically rational use of antimicrobials in emerging economies such as Cambodia
Daowood, et al. (2015) [15]	Malaysia	To evaluate children’s beliefs about medicines among primary schoolchildren and to explore their general knowledge about medicines	842 children (11 and 12 years old) and 842 parents of children included in the study	Cross-sectional survey Self-administration questionnaire (separate questionnaire for children and for parents)	Children’s age, gender and race affected their beliefs about efficacy of medicines. Females were more aware of the efficacy of medicine in comparison to males. This may be attributed to factors such as previous experience with medicine. Also, there is a possibility that girls have tendency to obtain more information from healthcare professionals. *Recommendations*: Educating children about medicines through the health school curricula. Healthcare professionals should play an active role to educate children. Qualitative research, in addition to quantitative research, to explain children’s expectations and practices related to medicine use.
Dong, et al. (2020) [16]	Vietnam	To describe the prevalence and antimicrobial susceptibility of commensal Neisseria from the oropharynx of a population with frequent antibiotic exposure.	207 self-identified men who have sex with men at least 18 years of age	Standardized questionnaire & collected 1 pharyngeal swab of both tonsillar pillars and the posterior oropharynx per participant	There is a lack of research on antibiotic usage behaviour of men who have sex with men. They often used antibiotics without prescription. This may be because of mistrust in confidentiality of healthcare providers due to stigma related to sexual orientation and a congested healthcare system. *Recommendations*: Stronger understanding of how antibiotic resistance is acquired in Neisseria gonorrhoeae
Fatokun (2014) [3]	Malaysia	To examine the pattern of antibiotic use and practices among individuals in a Malaysian community and to identify the variables associated with the likelihood of non-compliance with a course of antibiotic treatment	250 people ≥ 16 years of age	Cross-sectional survey Interviewer-administered questionnaire	Male gender, lack of knowledge of antibiotic functions, and lack of awareness of antibiotic resistance were significantly associated with a greater likelihood of non-compliance. Higher rate of non-compliance was observed among males than females (56.8% vs 44%). This may be due to females being more likely to seek help and visit healthcare facilities where they receive counselling on medication and health needs, thus making them more compliant *Recommendations*: Patient education and counselling about antibiotics and antibacterial resistance to enhance compliance to antibiotic therapy. Pharmacists should educate consumers on antibiotic resistance, compliance and proper disposal of unused antibiotics
Sato, et al. (2018) [17]	Philippines	To reveal fathers’ roles and perspectives with respect to the selection of care and treatment for children with pneumonia in a remote island of the Philippines	12 fathers whose children had pneumonia-like episodes in the 6 months prior to interview	Semi- structured interviews	Fathers take responsibility for caring for their sick children and made treatment decisions. Although fathers helped care for their sick children, mothers were the main caregivers. Fathers selected a treatment based on experience and beliefs, perceptions of the severity of the illness and financial burden. Fathers used antibiotics to treat coughs. Although fathers used antibiotics, they did not know their names or dosage. Mothers handled these aspects. *Recommendations*: Health education programmes to increase fathers’ knowledge and make the healthcare system more trustworthy to fathers. Healthcare providers need to understand fathers’ roles and perspectives when formulating health education programmes. It is also crucial to consider cultural background such as local beliefs. It is imperative to address issues related to medical cost and the credibility of health facilities to improve fathers’ healthcare-seeking behaviours.
Chanvatik, et al. (2019) [18]	Thailand	To assess the prevalence of antibiotic use, clinical indications and sources; knowledge and access to information related to antibiotics and AMR; and factors related to level of knowledge and access to information among Thai adult population	27,762 adults aged 15 years or above	Cross-sectional Structured interview questionnaire	Females, those older in age, those with higher education level and in richer wealth quintiles were more likely to receive public information about the appropriate use of antibiotics and AMR. Females have 1.18 times higher chances of receiving information on antibiotics than males. *Recommendations*: There should be policies which limit easy access to antibiotics. Population groups that should be targets for public campaigns are individuals with low education, members from poor households, men and younger persons.
Pham-Duc, et al. (2019) [19]	Vietnam	To explore knowledge, attitudes, and practices amongst livestock producers to identify their perspectives on antibiotic use and resistance.	392 small- & medium-scale producers specialized in pig, poultry and aquaculture production participated in the study (131 pig producers, 127 poultry producers, and 134 aquaculture producers)	Cross-sectional Knowledge, attitudes and practice questionnaire	Higher levels of knowledge and favorable attitudes about the use of antibiotics correlated with better reported practices when using antibiotics. Demographic information of the participants influences proper use and attitudes toward antibiotic use and antibiotic resistance. Most producers interviewed were male (71%). When it came to self- reported practices, female producers reported better practices than male producers. *Recommendations*: Initiatives to reward responsible prescription and distribution of antibiotics or promote antibiotic stewardship at the community level. Interventions should be context specific. Educational campaigns to improve producer awareness and removal of antibiotics from all commercially produced feed
Barber, et al. (2017) [20]	Philippines	To identify sociodemographic, knowledge and attitudinal correlates to antibiotic sharing among a community-based sample of adults (age 18 and older) in a low-income setting of the Philippines and to explore community-level data on informal antibiotic distribution in roadside stands	278 adults ≥18 years of age	Cross-sectional study Self-administered questionnaires	Contrary to previous research which indicated that being female was associated with antibiotic self-medication and non-antibiotic prescription drug sharing, in this study, antibiotic sharing was not associated with sociodemographic characteristics, including sex, or antibiotic knowledge. *Recommendations*: multipronged and tailored approaches to reduce informal antibiotic access
Ha, et al. (2019) [21]	Vietnam	To explore the awareness of antibiotic use and resistance among general people in highland provinces in Vietnam and detect associated factors.	1000 people from 1000 households ≥ 18 years, head of households or those who were responsible for purchasing medicines for the household	Cross-sectional study Structured Questionnaire	Females had a lower likelihood of being aware of prescription medicine compared to males Females, low education, low income, ethnic minorities, and those working in agriculture/fishery/forestry sector should be the target groups in future interventions in this setting. *Recommendations*: Educational interventions targeting females, those with low education, low income, belonging to ethnic minorities, and working in agriculture/fishery/forestry sector to improve awareness of antibiotic use and resistance
Aslam, et al. (2020) [22]	Malaysia	To validate and develop an instrument in Bahasa Melayu to assess the awareness and practices towards SMA in the Malaysian population.	100 adults 21–60 years of age	Pilot study to test validity and reliability of instrument	Knowledge and understanding scores differed significantly based on gender, education, duration of stay in Malaysia and ethnic group. Males had lower total knowledge and understanding about antibiotic use and antibiotic resistance in comparison to females. Practice scores for self-medication differed significantly based on gender, household income, and education. Females were found to be more prone to self-medicate compared males. *Recommendations*: The tool can be used to measure knowledge and practices and help develop effective educational interventions
Nepal & Bhatta (2018) [10]	WHO Southeast Asia Region (SEAR)	To quantify the frequency and effect of self-medication with antibiotics	19 full-text articles	Systematic Review	Prevalence of self-medication was higher among men in most studies. Reasons for this and further gender differences in antibiotic use not discussed in article. *Recommendations*: Educational interventions targeting the general public, pharmacists, and healthcare students. Improvement in the quality of healthcare facilities with easy access, law enforcement, and control regulations regarding the inappropriate use of antibiotics.

The main findings from these articles could be categorized into gender differences in: (i) knowledge of antibiotic use and AMR and (ii) practices related to antibiotic use. Gender differences in attitudes towards antibiotics were not discussed in depth in the included studies and thus attitudes have not been included as a theme in this review. None of the studies included models to examine the role of gender in antibiotic use.

A majority of the studies focused on knowledge of and practices related to antibiotics in humans [3, 14–18, 20–22]. One article in specific focused antibiotic use practices among men who have sex with men [16]. Three studies examined antibiotic use in animals, particularly in farms [1, 5] or among small- and medium-scale producers [19] and discussed potentials pathways for transmission of resistant bacteria from animals to humans including environmental pathways. None of the included studies focused primarily on gender differences in antibiotic use in plants and thus, antibiotic use in animals and plants have been combined as a sub-theme in the findings.

As some of the included studies discussed antibiotic resistance specifically whereas other referred to AMR in general, for the purpose of this scoping review, the term AMR.

### Knowledge of antibiotics and AMR

Knowledge of antibiotics and AMR referred to an understanding of the purpose of antibiotics [3, 14, 19], their efficacy [14, 15], the importance of adherence to antibiotic treatment [14, 20], appropriate use [18], sources of antibiotics (prescription and non-prescription) [10, 21] and AMR [3, 19, 21, 22].

#### Knowledge of antibiotics and AMR in humans

Results on gender differences in knowledge of antibiotics varied by context. However, generally, in studies examining antibiotic use in humans, females were found to have better knowledge of antibiotics and AMR in comparison to males. Some studies indicated that males had lower levels of knowledge, were more likely to believe that antibiotics could be used to treat viral infections and that adherence was not required once symptoms disappeared [14, 15, 22]. This was attributed to females being more likely to seek help and visit healthcare facilities, thus receiving more information on antibiotics [3, 15]. One study in particularly stated that women with higher levels of education and of higher socioeconomic status had 1.18 times higher chance of obtaining information on appropriate use of antibiotics and AMR in comparison to males [18]. A study among children in primary school examining knowledge of efficacy of medicines (including antibiotics) also found that young girls were more aware of the efficacy of medicine than boys and attributed this to girls having a greater tendency to obtain information from healthcare professionals [15]. When examining the roles of fathers and mothers in caring for children, results indicated that although fathers used antibiotics to treat symptoms such as coughs, mothers had greater knowledge of antibiotics due to being the primary caregivers and thus, having better health-seeking behaviour [17].

#### Knowledge of antibiotics and AMR in animals and plants

On the contrary, a study conducted among small-scale pig farms in Cambodia found that 74% of male farmers had heard of AMR in comparison to 39% of females. This was attributed to males having higher levels of education and being held responsible for treating sick pigs, thus resulting in more experience with antibiotics [5]. Similarly, a study examining awareness of prescription medicines including antibiotics also indicated that females had lower knowledge than males [21]. This was particularly found to be true among females who had lower levels of education, lower income, belonged to ethnic minority groups and worked in agriculture, fishery, or forestry [21].

When examining knowledge of antibiotic use and AMR in terms of environmental impact, one study on livestock and aquaculture producers found that a majority of producers agreed that AMR has a negative impact on the environment. However, differences in knowledge on this topic between genders was not examined [19].

Thus, although a greater number of studies included in this review indicated that females generally had greater knowledge of antibiotics in comparison to males, this was primarily true when examining antibiotic use in humans. Findings from articles on gender differences in knowledge of antibiotic use in animals and plants, however, contradicted this and found that males had greater knowledge. Thus, the relation between gender and knowledge of antibiotics remains unclear. This is due to knowledge on antibiotics being heavily related to gender norms and roles that exist within the community and thus varying between communities. Thus, there is a need to develop a clear understanding of contextual factors such as gender norms and roles that exist within a community of interest and how these factors influence knowledge of antibiotics and AMR prior to development and implementation of interventions to reduce inappropriate antibiotic use.

### Practices related to antibiotic use

Inappropriate antibiotic use was the primary focus of articles examining antibiotic use practices. Inappropriate antibiotic use referred to the use of leftover antibiotics [14], non-adherence to antibiotic treatments [3, 14], purchase and use of antibiotics without a prescription [14], antibiotic sharing practices [20] and self-medication [10, 22].

#### Practices related to antibiotic use in humans

Higher levels of knowledge were found to be associated with better practices related to antibiotic use [19, 20]. Males who had not heard of AMR were more likely to use leftovers or not complete an entire course of antibiotics [3, 14]. This was found to be particularly true among males with lower levels of education [14]. Adherence to antibiotic treatment was attributed to better health-seeking behaviour of females, making them more likely to receive counselling on medication from healthcare practitioners and thus, more compliant [3].

A review examining self-medication practices across Southeast Asia found that there were also higher levels of self-medication with antibiotics among males in comparison to females [10]. However, reasons attributing to the greater prevalence of such behaviour among males were not explored or discussed [10].

One study specifically focused on sexual orientation and examined the behaviour of men who have sex with men with regards to obtaining a prescription prior to taking antibiotics. This study found that men who have sex with men were less likely to seek healthcare services and get a prescription. This may be due to stigma associated with sexual orientation, a lack of trust in confidentiality being maintained by healthcare providers, and a crowded healthcare system [16].

#### Practices related to antibiotic use in animals and plants

With regards to antibiotic use in animals, antibiotics were highly used for prophylactic purposes as opposed to for treatment, thus resulting in increased risk of resistance [1]. Females were found to have better practices related to antibiotic use in animals in both studies on this topic included in this review [1, 19]. Reasons for this, however, were not explored in the studies. None of the included studies examined gender differences in practices related to antibiotic use in plants.

Similar to studies examining knowledge of antibiotics and AMR, there were inconsistencies among the findings of studies examining antibiotic use practices in humans, animals and plants. Although most studies indicated that females had better practices related to antibiotic use, one study found that despite males having lower knowledge of antibiotic use and resistance, females were more prone to self-medicate [22]. Moreover, a study which particularly focused on antibiotic misconceptions and non-medical access at the community level found no association between gender and self-medication with antibiotics. Nonetheless, it is important to note that the authors of this study stated that their findings were contrary to previously conducted research which indicated that being female was associated with self-medication with antibiotics [20].

Overall, when examining the role of gender in knowledge and practices related to antibiotics, it is evident that further exploration of reasons for gender differences in these areas is required. Findings on these topics are scarce and appear to vary by context. In addition, gender was often discussed in intersection with other sociodemographic characteristics, particularly level of education and socioeconomic status. Level of education and socioeconomic status were the most commonly used criteria for sociodemographic characteristics in the included studies from different countries across Southeast Asia. Thus, further analysis of the influence of gender on antibiotic knowledge and use, and the intersectionality of gender with other sociodemographic factors that influence inappropriate antibiotic use, must be examined carefully in order to strengthen AMR surveillance strategies at the local and national levels.

### Recommendations

Targeted educational interventions and further qualitative research were provided as key recommendations for next steps in relation to reducing inappropriate use of antibiotics and thus, minimizing the impact of AMR [1, 14, 15]. Gender plays an important role in these recommendations and the intersectional nature of gender with education levels, socioeconomic status and other sociodemographic characteristics must be considered when developing future interventions or conducting further research.

#### Educational interventions

Although findings on knowledge of males and females were not consistent among the articles included in this review, generally a need to improve knowledge on antibiotic use and AMR among the general public was identified [1, 14, 15]. Improved knowledge will in turn promote better practices related to antibiotics, thus mitigating risk of resistance in humans, animals and the environment. As levels of existing knowledge and current practices vary by gender, there is a need for educational interventions to be targeted and designed with a good understanding of how gender norms and roles in the community influence antibiotic use. For instance, if females are seen as the primary caregivers in a specific community and responsible for treating family members when ill, or in the case of farms, sick animals, identifying females as entry points to improving antibiotic use in this community and creating messages in a manner relatable to their roles in the community will prove to be effective.

These interventions need to focus not just on raising awareness of antibiotic use but also AMR as well [3, 18] with the aim that greater understanding of AMR and its consequences will reduce inappropriate practices such as non-adherence, use of antibiotics as prophylactics and growth promoters, antibiotic sharing and self-medication. Farmers should specifically be trained on appropriate administration of antibiotics to animals and the impact of high levels of antibiotic residue in animals for consumption on humans as well as pathways for transmission of resistance [1].

As the main source of antibiotics was found to be pharmacies, pharmacists play a key role in educating the general public [3, 10]. In addition, healthcare professionals should be actively involved in the development of educational interventions to promote health-seeking behaviour and better antibiotic use practices among the public [15, 22]. Studies also indicated that information on antibiotic use and AMR should be integrated into school curricula as misconceptions on efficacy of medicine exist even among school children and vary by gender [14, 15]. Raising awareness at a younger age could result in promotion of good practices related to antibiotic use early, thus reducing the risk of resistance.

Targeted educational interventions aimed at reducing inappropriate antibiotic use specifically in animals should provide information on animal healthcare, specific approaches to treating different animals such as livestock and aquaculture, the effects of inappropriate use pertaining to the specific animals and how inappropriate use can be avoided [19]. This is especially important as the approaches for care and use of antibiotics vary by animal [19]. A study conducted in Vietnam examining knowledge, attitude and practices of livestock and aquaculture producers regarding antibiotic use and AMR not only found gender differences in antibiotic use, but also found significant differences in knowledge, attitudes and practices between pig producers, poultry producers and aquaculture producers [19]. This further emphasizes the need for educational campaigns to be targeted to the different types of producers with context-specific information pertaining to different animals [19].

#### Further qualitative research

Targeted interventions require in-depth understanding of factors that influence knowledge and use of antibiotics among males and females. As research on gender and antibiotic use is limited, and research exploring reasons for gender differences in knowledge and practices related to antibiotics even more scare, there is a need for further qualitative research to develop a greater understanding of differences in knowledge and practices between genders and underlying reasons for these differences. For instance, when exploring the behaviour of farmers in the Mekong Delta of Vietnam, it was found the female farmers were less likely to use antibiotics, however explanations for this behaviour were not explored [1]. To promote appropriate antibiotic use, reasons for such differences in behaviour need to be identified and addressed. Moreover, as these factors are context specific and often intersect with other sociodemographic characteristics, there is a need for further qualitative research within the communities of interest when developing interventions. It is important to note that such qualitative research should be conducted to complement quantitative research aimed at identifying existing knowledge, attitudes and practices related to antibiotic use and effects of misuse [15]. Conducting gender analysis research prior to developing interventions and continuously assessing the impact of the interventions by gender will be beneficial and improve the effectiveness of strategies to reduce the impact of resistant bacteria.

#### Other recommendations

Inappropriate use of antibiotics through practices such as self-medication are found to be associated with factors such as inaccessibility, unregulated distribution of medicines and lack of medical professionals and quality healthcare facilities, to name a few [10]. Efforts must be taken at all levels to prevent such inappropriate practices [10]. Governments across ASEAN have developed national action plans to monitor and regulate the use of antibiotics [6, 19]. Despite the existence of such national action plans, there remains a need to further strengthen existing policies in order to effectively monitor and regulate antibiotic use [6]. Indicators measuring factors such as equity and accountability are not the same across countries in the region [6, 19]. For instance, although it is recommended that gender and socioeconomic considerations be made when promoting equity with regards to AMR, not all countries in ASEAN include socioeconomic considerations in their action plans and none of the countries have included gender considerations [6]. Reliable indicators on antibiotic use and AMR in agriculture is particularly limited across the region despite the large use of antimicrobials in this sector [19]. Having regional indicators to monitor and regulate AMR in addition to context-specific indicators will prove to be beneficial in tackling AMR through a One Health approach.

Inappropriate use of antibiotics results in increased risk of mistreatment, adverse drug reactions and resistance [10]. These factors along with the higher burden of infectious diseases in Southeast Asia put healthcare systems in the region more at risk of financial strain [10]. The establishment and enforcement of laws and regulations aimed at ensuring appropriate dispensing of antibiotics through pharmacies as well as increasing awareness of the adverse effects of inappropriate use, quality of antibiotics and promoting health-seeking behaviour can mitigate the risk of AMR [10].

In addition, the high costs associated with hospital treatment affects health-seeking behaviour [17], thus resulting in people opting to use low cost and easily available antibiotics such as amoxicillin [10]. A study conducted examining father’s roles in healthcare seeking for their children found that providing more flexible and affordable options for payment improved health-seeking behaviour as was the case with traditional healers in the Philippines [17]. Implementing regulations to improve affordability of healthcare and provide flexible payment options could result in reduced inappropriate use of antibiotics and promote health-seeking behaviour as well.

A study examining the effects of self-medication with antibiotics across Southeast Asia highlighted the need for organizations at the regional level such as the World Health Organization (WHO), the South Asian Association for Regional Cooperation (SAARC), the Association of Southeast Asian Nations (ASEAN), and the Ministry of Health of countries in the region to work together in the development of interventions targeting common inappropriate use practices across the region [10].

Furthermore, although efforts are being made to reduce inappropriate use and monitor AMR, these efforts focus primarily on human health [19]. There is a need for more data and regulations focusing antibiotic use and resistance in animals and the environment [19]. The national action plans of all ASEAN countries emphasize the importance of One Health engagement, in other words engagement of stakeholders at all levels (decision-makers, regulatory authorities, medical practitioners, veterinarians, pharmacists, etc.) and across human, animal and environmental health sectors, in order to effectively tackle AMR [6]. However, efforts focusing on animal health were found to be scarce, with those aimed at environmental health even more limited [6, 19]. Thus, there is a need to strengthen multisectoral efforts in order to reduce inappropriate antibiotic use in human, environmental and animal health and tackle AMR across nations.

When examining laws and regulations, it is especially important to examine the availability and use of over-the-counter antibiotics as well as the quality of antibiotics. Antibiotics are found to be readily available in local shops across Southeast Asia, as was evident in the study examining the role of roadside (sari-sari) stands in antibiotic distribution in the Philippines [20]. Sari-sari stands are small businesses commonly found in residential areas and observation of these stands indicated that many of them carried and distributed antibiotics that were expired or did not contain an expiration date, thus posing health concerns due to poor quality of these medicines [20]. Interventions at the national as well as the local level are needed to regulate the distribution of antibiotics and particularly reduce the distribution of low-quality antibiotics in countries across Southeast Asia [20]. The quality of antibiotics was usually discussed in terms of purchase without prescription as opposed to the quality of prescribed antibiotics in the included studies. However, the need for people to be aware of quality and check details of the antibiotics they purchase was recommended.

Similarly, when examining antibiotic use among animals in Vietnam, legal antibiotics were often found in livestock feed along with illegal drugs [19]. Removal of antibiotics in commercial feed products is required to combat AMR in animals [19]. In addition, antibiotics were also found to be commonly used for prophylactic reasons and as growth promoters [19]. This inappropriate use of antibiotics in animals may be attributed to the availability of over-the-counter antibiotics and limited supervision by veterinarians, thus resulting in antibiotic residue in meat products which in turn could affect human health and the environment [19]. A Law on Animal Husbandry was developed by the Vietnamese government in order to tackle AMR and antibiotic residues in livestock. However, regulation has proven to be difficult, particularly among smallholder farms, thus emphasizing the need for more regulation of such antibiotic use in animals [19].

In addition to laws and regulations promoting management of quality antibiotics, rewarding responsible prescription and distribution at the community level will prove to be effective in tackling inappropriate antibiotic use and AMR at the community level [19].

## Conclusion

Inappropriate antibiotic use is a major contributor to AMR particularly in Southeast Asia. Although studies indicate an association between sociodemographic factors, such as gender, and knowledge and practices related to antibiotic use, research is limited. This scoping review aimed to map out existing evidence on gender differences in antibiotic use in Southeast Asia. Research indicates that better knowledge of antibiotic use and AMR are associated with better practices. However, whether males or females have more knowledge and better practices was found to be highly contextual and dependent on other sociodemographic factors. Thus, generalizations on gender differences cannot be made. Despite findings being inconsistent, most studies included in this review did indicate that gender differences do in fact exist. However, reasons for these differences between genders require further qualitative research at the community level.

Targeted educational interventions were recommended in most studies as the best way to inform, and thus reduce, inappropriate antibiotic use. Healthcare settings and healthcare professionals were seen as playing a vital role in these interventions to promote better use of antibiotics at the community level. Such interventions require further qualitative research on the influence that sociodemographic factors such as gender have on antibiotic use. Thus, conducting a gender analysis using both quantitative and qualitative methods to inform the design and implementation of interventions addressing AMR will result in better outcomes for males and females, not only leading to better antibiotic use behaviour but also promoting gender equity within communities by addressing the specific needs of the most marginalized. In addition, such research and interventions will inform and strengthen existing AMR surveillance strategies.

In addition to the two aforementioned recommendations, other recommendations particularly focusing on cost, quality, distribution, and development of reliable indicators were also suggested as necessary to improving monitoring and regulation of antibiotic use and AMR. These recommendations require strengthening of policies, laws and regulations through the involvement of stakeholders from the regional, national and local levels and collaboration between the domains of animal, human and environmental health.

It is important to note that the study populations of the articles included in this review varied greatly by size, age and gender, which may put to question the justification of this conclusion. As this review aimed to map out any existing research on gender differences in antibiotic use, similarity in study population was not a criterion for inclusion. Whether males or females have more knowledge of and better practices related to antibiotic use and AMR was found to be highly contextual and dependent on other sociodemographic factors in addition to gender, thus emphasizing the need for qualitative research specifically at the community level and targeted educational interventions designed based on a solid understanding of localized needs.

In addition, there was also variability in the research design of the included studies. Most of the studies involved self-administered and interviewer-administered questionnaires, thus leaving room for self-reporting and recall bias. However, a majority of these studies reported having taken measures to ensure validity and reliability such as pilot testing, verifying questionnaires with experts, training of data collectors, and summarizing of findings with the participants to ensure accurate understanding, thus justifying the conclusions outlined in these studies. Also, despite studies outlining self-reporting bias as a limitation due to dependency on the respondent’s ability to recall and level of comprehension, the authors highlighted that the findings served as a starting point for further exploration when discussing such limitations, further reiterating the need for more research on the topic.

This review has some limitations. Only English language articles were considered for inclusion in this review. This presents as a limitation as exploration of Vietnamese language literature may have provided more details on gender and antibiotic use specifically within the context of Vietnam. In addition, only open-access articles were screened and included in this study. Thus, this presents a possibility for important information in articles that are not available for free to be missed. However, to mitigate the risk of this, two searches were conducted on multiple databases and the searches continued until a point of saturation was reached (i.e. until search results contained many duplicates across databases). Despite these limitations, this review is the first of its kind, emphasizes the need for further exploration and serves as a starting point for further research.

## Supporting information

S1 File PRISMA checklist. (DOC)

## References

[1] Carrique-Mas JJ, Trung NV, Hoa NT, Mai HH, Thanh TH, Campell JI, et al. Antimicrobial usage in chicken production in the Mekong Delta of Vietnam. Zoonoses Public Health. 2015;62(1):70–8. 25430661 10.1111/zph.12165

[2] Hedman HD, Vasco KA, Zhang L. A review of antimicrobial resistance in poultry farming within low-resource settings. Animals. 2020;10(1264). doi: 10.3390/ani10081264 32722312 PMC7460429

[3] Fatokun O. Exploring antibiotic use and practices in a Malaysian community. Int J Clin Pharm. 2014; 36(3):564–9. doi: 10.1007/s11096-014-9937-6 24700341

[4] Holloway KA, Kotwani A, Batmanabane G, Puri M, Tisocki K. Antibiotic use in South East Asia and policies to promote appropriate use: reports from country situational analyses. BMJ. 2017;358:9–13. doi: 10.1136/bmj.j2291 28874360 PMC5598252

[5] Strom G, Boqvist S, Albihn A, Fernstrom LL, Andersson Djurfeldt A, Sokerya S, et al. Antimicrobials in small-scale urban pig farming in a lower-middle income country–arbitrary use and high resistance levels. Antimicrob Resist Infect Control. 2018;7(35). doi: 10.1186/s13756-018-0328-y 29541447 PMC5842516

[6] Chua AQ, Verma M, Hsu LY, Legido-Quigley H. An analysis of national action plans on antimicrobial resistance in Southeast Asia using a governance framework approach. Lancet Reg Health West Pac. 2021;7(100084). doi: 10.1016/j.lanwpc.2020.100084 34327414 PMC8315476

[7] WHO. Tackling antimicrobial resistance (AMR) together–working paper 5.0: enhancing the focus on gender equity. Geneva, Switzerland. 2018. https://apps.who.int/iris/handle/10665/336977.

[8] Morgan DJ, Okeke IN, Laxminarayan R, Perencevich EN, Weisenberg S. Non-prescription antimicrobial use worldwide: a systematic review. Lancet Infect Dis. 2011;11(9):692–701. doi: 10.1016/S1473-3099(11)70054-8 21659004 PMC3543997

[9] Schmiege D, Evers M, Kistemann T, Falkenberg T. What drives antibiotic use in the community? A systematic review of determinants in the human outpatient sector. Int J Hyg Environ Health. 2020;226(113497). doi: 10.1016/j.ijheh.2020.113497 32120251

[10] Nepal G, Bhatta S. Self-medication with antibiotics in WHO Southeast Asia region: a systematic review. Cureus. 2018;10(4). doi: 10.7759/cureus.2428 29876150 PMC5988199

[11] Mackenzie JS, Jeggo M. The One Health approach–why is it so important? Trop Med Infect Dis. 2019; 4(88). doi: 10.3390/tropicalmed4020088 31159338 PMC6630404

[12] Peters MDJ, Godfrey C, McInerney P, Munn Z, Tricco AC, Khalil H. Chapter 11: scoping reviews (2020 version). In Aromataris E, Munn Z, editors. JBI manual for evidence synthesis. https://synthesismanual.jbi.global.

[13] cfr.org. [Internet]. What is ASEAN? [cited 2021]. https://www.cfr.org/backgrounder/what-asean.

[14] Choo SJ, Chang CT, Lee JCY, Munisamy V, Tan CK, Raj JD, et al. A cross-sectional study on public belief, knowledge and practice towards antibiotic use in Perak, Malaysia. J Infect Dev Ctries. 2018;12(11):960–9. doi: 10.3855/jidc.10723 32012125

[15] Daowood OT, Ibrahim MIM, Abdullah AC. Children’s knowledge and beliefs about medicines. J Child Health Care. 2015;19(1):73–83. doi: 10.1177/1367493513496911 23975718

[16] Dong HV, Pham LQ, Nguyen HT, Nguyen MXB, Nguyen TV, May F, et al. Decreased Cephalosporin Susceptibility of Oropharyngeal Neisseria Species in Antibiotic-using Men Who Have Sex With Men in Hanoi, Vietnam. Clin Infect Dis. 2020;70(6):1169–75. doi: 10.1093/cid/ciz365 31049592 PMC7319061

[17] Sato M, Oshitani H, Tamaki R, Oyamada N, Sato K, Nadra AR, et al. Father’s roles and perspectives on healthcare seeking for children with pneumonia; findings of a qualitative study in a rural community of the Philippines. BMJ Open. 2018;8(11). doi: 10.1136/bmjopen-2018-023857 30467133 PMC6252634

[18] Chanvatik S, Kosiyaporn H, Lekagul A, Kaewkhankhaeng W, Vongmongkol V, Thunyahan A, et al. Knowledge and use of antibiotics in Thailand: a 2017 national household survey. PLoS One. 2019;14(8). doi: 10.1371/journal.pone.0220990 31398242 PMC6688796

[19] Pham-Duc P, Cook MA, Cong-Hong H, Nguyen-Thuy H, Padungtod P, Nguyen-Thi H, et al. Knowledge, attitudes and practices of livestock and aquaculture producers regarding antimicrobial use and resistance in Vietnam. PLoS One. 2019;14(9). doi: 10.1371/journal.pone.0223115 31553776 PMC6760827

[20] Barber DA, Casquejo E, Ybanez PL, Pinote MT, Casquejo L, Pinote LS, et al. Prevalence and correlates of antibiotic sharing in the Philippines: antibiotic misconceptions and community-level access to non-medical sources of antibiotics. Trop Med Int. Health. 2017;22(5):567–75. doi: 10.1111/tmi.12854 28187247

[21] Ha TV, Nguyen AMT, Nugyen HST. Public awareness about antibiotic use and resistance among residents in highland areas of Vietnam. 2019;2019. doi: 10.1155/2019/9398536 31223624 PMC6541961

[22] Aslam A, Gajdacs M, Zin CS, Rahman NSBA, Ahmed SI, Jamshed SQ. Public awareness and practices towards self-medication with antibiotics among the Malaysian population. A development of questionnaire and pilot-testing. Antibiotics (Basel). 2020;9(2). doi: 10.3390/antibiotics9020097 32102325 PMC7168161

